# Wearable Frontal EEG for Screening Cognitive Impairment

**DOI:** 10.1002/alz.090080

**Published:** 2025-01-09

**Authors:** Nayoung Ryoo, SangYun Kim, Young Gyu Lee, Yongwook Chae

**Affiliations:** ^1^ Eun‐pyeong St. Mary’s Hospital of the Catholic University of Korea, Seoul, Seoul Korea, Republic of (South); ^2^ Seoul National University Bundang Hospital, Seoul National University College of Medicine, Seongnam Korea, Republic of (South); ^3^ Looxid Labs, Seoul, Seoul, Seoul Korea, Republic of (South); ^4^ Looxid Labs, Seoul, Seoul Korea, Republic of (South)

## Abstract

**Background:**

Alzheimer's disease (AD) is a neurodegenerative disorder that is characterized by progressive cognitive decline. Early detection of AD is essential for timely intervention and treatment. The objective of this study is to explore the potential usefulness of virtual reality (VR) tasks combined with frontal EEG recordings for early detection of cognitive impairment in individuals with SCD (subject cognitive decline), MCI (mild cognitive impairment), and AD (Alzheimer's disease). We aimed to analyze the results and compared them with those of a control group with normal cognition.

**Method:**

Resting‐state EEG was recorded in the awake‐resting state with closed eyes, and task‐induced EEG recordings were obtained during VR tasks from 108 participants. The participants included those with SCD, MCI, and AD, and they were compared with 18 subjects with normal cognition. Alpha reactivity was determined by measuring the decrease in relative alpha power over frontal electrodes.

**Result:**

This study observed a significant decrease in alpha reactivity in the order of SCD, MCI, and AD compared to the control group with normal cognition in EEG recordings (P‐value < 0.05). Additionally, the decibel power ratio of the EEG recorded during cognitive tasks exhibited a reversed order of decrease compared to the resting state across the same diagnostic groups.

**Conclusion:**

This study shows that when performing cognitive tasks with a virtual reality (VR) device on the forehead, distinct features for Alzheimer's disease are observed compared to the healthy control group. While larger and longer‐term studies are necessary, these findings suggest that cost‐effective digital biomarkers using measurable EEG could be valuable for early Alzheimer's diagnosis.

